# A case of atypical infantile de novo antiphospholipid syndrome presenting with neonatal ischemic stroke without any triggering risk factors as a “second hit” and review of the literature

**DOI:** 10.1007/s12026-025-09605-w

**Published:** 2025-02-19

**Authors:** Doruk Bor, Mustafa Vakur Bor

**Affiliations:** 1 Department of Clinical Biochemistry, University Hospital of Southern Denmark, Finsensgade 35, 6700 Esbjerg, Denmark; 2 Faculty of Medicine, Aarhus University, Aarhus, Denmark; 3 Thrombosis Research, Department of Regional Health Research, University of Southern Denmark, Esbjerg, Denmark

**Keywords:** De novo, Atypical, Infantile antiphospholipid syndrome, Thrombotic risk factors, Ischemic stroke

## Abstract

Infantile antiphospholipid syndrome (APS) is a rare condition arising from either transplacental transfer of antiphospholipid antibodies (aPL) from the mother or de novo antibody production in the newborn. We present a unique case of cerebral artery thrombosis with persistently elevated anti-cardiolipin and anti-β2-glycoprotein-I antibodies, despite the absence of maternal aPL, suggesting primary de novo aPL synthesis. While the prevailing “second-hit” hypothesis suggests that additional thrombotic risk factors are required to trigger APS in infants, our case exhibited no prenatal, maternal, or thrombophilic risk factors. A literature review of 20 reported cases further confirmed that ours was the only case without additional thrombotic triggers among the 18 cases with complete data, challenging the necessity of a “second hit” in neonatal APS. Notably, aPL levels normalized over time without recurrence, raising questions about the need for long-term anticoagulation in select cases, including ours. These findings suggest a potential transient form of infantile APS and highlight the importance of sequential aPL testing to guide treatment. Further research is required to elucidate the mechanisms underlying de novo aPL synthesis and its clinical implications.

## Introduction

Antiphospholipid syndrome (APS) in adults is a well-established condition characterized by the occurrence of arterial, venous, or both, thrombosis, along with pregnancy morbidity, all accompanied by consistently positive antiphospholipid antibodies (aPL). These antibodies include lupus anticoagulant (LA), anti-cardiolipin (aCL), and/or anti-β2-glycoprotein I antibodies (aβ2GPI). Diagnosis typically requires the detection of these antibodies on a minimum of two consecutive occasions at least 12 weeks apart, in accordance with the international (Sydney) consensus statement criteria [1]. However, for research purposes, classification criteria were revised by the American College of Rheumatology (ACR) and the European League Against Rheumatism (EULAR) in 2023 which introduced a positive aPL test as an entry criterion and applied additive weighted criteria across clinical domains for APS classification [2].

Despite the comprehensive characterization of APS in adults, the literature currently lacks reliable data on the incidence and prevalence of pediatric APS due to the absence of validated clinical criteria [3]. Presently the diagnosis in the pediatric population relies on the application of adult criteria along with clinical assessment.

Infantile APS is a rare condition and only a few cases are reported in the literature [4–7]. The etiology of infantile APS can be classified into two primary factors: the transmission of maternal pathogenic antibodies through the placenta and the de novo synthesis of antibodies by the infant which is especially rare [8]. Evidence suggests that de novo infantile APS may necessitate the presence of additional thrombotic risk/trigger factors, often referred to as the “second-hit” mechanism as illustrated in Table 1 [4–8].

**Table 1:** Additional trigger/risk factors reported in the literature as a “second hit mechanism” associated with the onset of thrombotic events in infantile APS

Prenatal	Maternal	Hereditary thrombophilia
Prematurity	Gestestainal diabetes mellitus	Antithrombin deficiency
Catheters	Abruptio placentae	Protein-C deficiency
Infection/sepsis	Preeclampsia	Protein-S deficiency
Prenatal asphyxia	Infection	Factor-V Leiden mutation
Respiratory distress	Intrauterine growth restriction	Prothrombin G20210A mutation
Fetal inflammatory response syndrome	Antiphospholipid syndrome	Methyltetrahydrofolate reductase mutations
Parenteral nutrition	Hereditary thrombophilia	Plasminogen activator inhibitor-1 mutations
Dehydration	Twin pregnancy	
Cardiac disease	Recurrent pregnancy losses	
Macrosomia	Intrauterine fetal death	
Acute kidney injury	Thrombus in the umbilical artery or vein	
Chylothorax		
Hypoxic ischemic encephalopathy		

In the present paper, however, we report a case of atypical infantile de novo APS presented with a neonatal stroke, without the presence of additional risk/trigger factors, and discuss the treatment approach of this case. We further review the literature focusing on the additional risk factors related to reported cases with de novo infantile APS.

## Materials and method

We performed a search of the literature for cases of infantile APS by using the keywords “neonatal” or “perinatal” or “infantile” together with “antiphospholipid antibody or syndrome.” in the electronic databases Medline/Pubmed, Embase, Scopus, and Directory of Open Access Journals through 9th of January 2025. The literature search was supplemented by a manual review of the references in the identified publications. We primarily reviewed English-language abstracts and articles, considering all study designs and article types, including case reports and letters to the editor, but excluding congress or meeting abstracts. Among this literature search, cases fulfilling the de novo occurrence of APS on the following criteria included this review: (a) neonatal, perinatal, and infantile presentation of thrombosis; (b) persistent positive aPL antibodies, including LA (analyzed on two different occasions); and (c) negative aPL antibodies, including LA in the mother or cord blood.

Study design, sex of the patients, clinical presentation, localization of thrombosis, infant and maternal aPL status, maternal, prenatal, thrombophilia risk factors, and anticoagulation treatment were extracted from the articles. The data are summarized in Table 2.

**Table 2:** Clinical and laboratory presentation, maternal, prenatal, and thrombophilic risk factors, as well as anticoagulation (AC) treatment strategies for patients with de novo infantile APS reported in the literature

Patient nr	Reference/study design	Clinical presentation (age at symptoms)	Localization of thrombosis (investigation time)	Infant aPL (testing time)	Maternal aPL	Maternal risk factors	Prenatal complication/risk factors	Thrombophilia risk factors	AC treatment
1. Male	Alshekaili et al. (2010) [9]; case report	Reduced movement of right limbs (1 month) (5 days)	Ischemic stroke of the left middle cerebral artery (6 months)	aβ2GPI-IgG (6 months); aCA, IgG (7 months)	Negative	None	None	Heterozygosity FV Leiden mutation	NA
2. Male	Cabral et al. (2011) [10]; case report	Symptoms for necrotizing enteritis complicated by cardiac and renal failure (17 days-6 months), two different episodes of ischemic stroke (13 months) diagnosed as catastrophic APS	Small bowl infarction(17 days), renal vein thrombosis (6 months) infarct at the left frontal, temporal, and parietal lobes (13 months)	LA (2 years); aCA-total, aβ2GPI Ig G (13 months)	Negative	None	Infection (positive for *E. coli* in blood); septic shock; prematurity; central catheter	Prothrombin (20210G/A) mutation PAI-1 (675G/A) gene mutation	ASA and LMWH
3. Male	20 Merlin et al. (2012) [11]; case report	Right hemicorporeal seizures (3 days)	Superficial territory of the left middle cerebral artery infarction (NA)	aCA IgG, aβ2GPI IgG (1 year)	Negative	Diabetes	Macrosomia	Lipoprotein(a) level of 41 mg/dl (*N* ≤ 30 mg/dl)	ASA
4. Female	Sousa et al. (2012) [12]; case report	Multifocal clonic seizures and left-sided hemiparesis (8 h)	Ischemic stroke at right caudate and lenticular nucleus with extension to the cerebral peduncle (NA)	LA, aCA IgG, aβ2GPI IgG, (3 months)	Negative	None	None	MTHFR Homozygous mutation (1298C/C), a double homozygous PAI-1 (844A/A and 675 4G/4G) mutations	ASA
5. Male	Gordon et al. (2014) [7]; case report and review of literature	Edema of the left leg, dehydration, thrombocytopenia. *E. coli* sepsis (9 days)	DVT in popliteal veins, common femoral vein extending up to the left external iliac vein (NA)	aCL IgG IgM, aβ2GPI IgM (16 days and 3, 5–6 months)	Negative	None	Sepsis, dehydration	None	UFH and later LMWH
6. Male	Berkun et al. (2014) [5]; case series (*n* = 12), including 8 in the present review	Hypertonia (7 months)	Infarct of basal ganglia and left parietal infarct (NA)	Persistent aPL^1^ (NA)	Negative	Repeated pregnancy losses, protein S deficiency	None	None	NA
7. Female		Dystonic hemiplegia (4 months)	Right median cerebral artery occlusion (NA)	Persistent aPL^1^ (NA)	Negative	None	None	NA	NA
8. Female^2^		Epilepsy, hemiparesis (3 months)	Left median cerebral artery stroke(NA)	Persistent aPL^1^ (NA)	Negative	None	None	Heterozygosity FV Leiden and prothrombin (20210G > A) mutations	NA
9. Male		Perinatal asphyxia, intracranial hemorrhage, seizure, hemiplegia (8 months)	Left parietotemporal stroke (NA)	Persistent aPL^1^ (NA)	Negative	Intrauterine fetal death, Thrombus in umbilical artery	Prematurity, asphyxia, Respiratory Distress Syndrome, Sepsis	None	NA
10. Female^2^		Hemiparesis (4 months)	Left median cerebral artery stroke (NA)	Persistent aPL^1^ (NA)	Negative	None	None	MTHFR (677C/T) Homozygous mutation	NA
11. Female		Seizure (3 days)	Infarct right median cerebral artery (NA)	Persistent aPL^1^ (NA)	Negative	NA	None	None	NA
12. Male		Lethargy, seizure (3 days)	Cerebral sinus vein thrombosis (NA)	Persistent aPL^1^ (NA)	Negative	None	sepsis	None	LMWH
13. Female		Seizure (3 days)	Left parietooccipital stroke (NA)	Persistent aPL^1^ (NA)	Negative	None	Prematurity, asphyxia, respiratory distress syndrome, sepsis	MTHFR (677C/T) homozygous mutation	NA
14. Female	Giani T. et al. (2020) [13]; case series (*n* = 4), including 2 in the present review	None	Frontotemporal parietal stroke presumed (NA)	aβ2GPI, IgG, IgM (23 months)	Negative	Gestational diabetes, twin pregnancy	Prematurity, respiratory distress syndrome	None	None
15. Female		Left hemiparesis (5 months)	Right middle cerebral artery stroke presumed (6 months)	aβ2GPI, IgG aCL, IgG (6 months)	Negative	Gestastional diabetes	None	None	None
16. Male		Jerking movements, hypotonia, partial seizures, poor spontaneous movements (10 days)	Left middle cerebral artery stroke presumed (NA)	aβ2GPI, IgG aCL, IgG (5 months)	Negative	Abruptio placentae	None	Heterozygosity FV Leiden mutation	ASA
17. Male	Bitsadze V. et al. (2020) [8]; updated the article with a case report	Acute respiratory distress syndrome, thrombocytopenia, died 21 days after the delivery due to massive thrombosis and multi-organ failure, diagnosed as catastrophic APS	Multiple thrombosis and catastrophic APS (NA)	aCA Ig M (NA)	Negative	Thrombus in the umbilical vein, *Escherichia coli* infection	Prematurity, respiratory distress syndrome, fetal inflammatory response syndrome	None	None
18. Female	Pines M. et al. (2023) [14]; 2 cases; letter to the editor	Respiratory failure, chylothorax, and UTI after ASD repair at 6 weeks old. Echo showed right atrial thrombus post-op	a right atrial thrombus (92 days)	aβ2GPI, IgG (NA)	Negative	None	Central venous catheter; residual valvulopathies; chylothorax; infection; acute kidney injury	None	LMWH
19. Male		Received respiratory support for thick meconium at birth. Day 1: seizure. Head imaging showed ischemia, hemorrhage, and thrombose	Thrombus in multiple cerebral veins, sinuses, and arteries, including the left internal carotid (5 days)	aβ2GPI, IgG (3 months)	Negative	None	Chorioamnionitis; asphyxia; hypoxic-ischemic encephalopathy	None	LMWH
20. Female	Bor et al. current report; case report and review	Reduced spontaneous movement in the left limbs (3.5 months)	Encephalomalacia, due to a result of a left middle cerebral artery infarction (5 months)	aβ2GPI, IgG, aCL, IgG (7.5 months)	Negative	None	None	None	ASA

*aPL:* antiphospholipid antibodies, *aβ2GPI:* anti-β2-glycoprotein I antibodies, *aCL:* anti-cardiolipin  antibodies, *ASD:* atrioventricular septal defect, *DVT:* deep vein thrombosis, *Echo:* echocardiogram, *FV Leiden:* factor V Leiden, *LMWH:* low-molecular-weight heparin, *LA:* lupus anticoagulant, *MTHFR:* methylenetetrahydrofolate reductase, *NA:* non-available. *PAI-1;* plasminogen activator inhibitor-1, *UFH:* unfractionated heparin, *UTI:* urinary tract infection

^1^The aPL profile for each patient is not specified in the publication, as the article primarily includes case series. However, it is noted that the patients had persistent aPL

^2^Patients #8 and #10 appear to have been published by the same author in 2006 (Berkun et al., *Arthritis & Rheumatism*, 2006; 55:850–855). Therefore, these cases from the 2006 publication were not included in Table 2

Information about our patient was retrieved from the electronic medical journal of the Thrombosis Clinic at the University Hospital of Southern Denmark, Esbjerg. Informed consent was obtained from the patient’s family to publish this paper.

LA was detected using a CS5100 coagulation analyzer (Sysmex Corporation, distributed by Siemens Healthcare) in accordance with ISTH guidelines [15] using two recommended assays with a CV of < 10%, the lupus-sensitive APTT, PTT-LA (Diagnostica Stago, Asnières-sur-Seine, France), and the dRVVT (Precision Biologic, Dartmouth, Canada). LA was considered positive if at least one of the two tests yielded a positive result.

The detection of aCL and aβ2GPI in the patient and patient’s mother was carried out using enzyme-labeled anti-isotype Assay (EliA) (Thermo Fisher Scientific), fully automated random access fluorescence enzyme immunoassays on the Phadia 250 instrument (Phadia GmbH) as previously described [16]. Both antibodies exhibited a CV of < 10%. A 99th percentile as the upper cutoff in the diagnosis of positive aPLs was utilized and determined through in-house validation in accordance with the approach suggested by the International Society on Thrombosis and Haemostasis (ISTH) [16].

### Case presentation

**Fig. 1:**
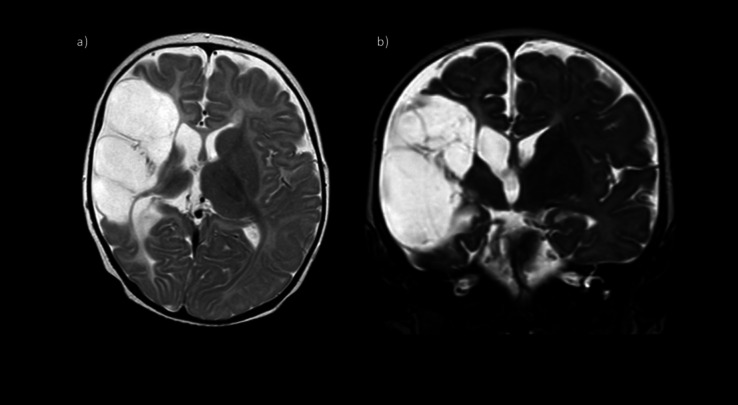
Axial (**a**) and coronal (**b**) T2-weighted images showing an extensive area of encephalomalacia involving the right frontal, temporal, and parietal lobes consistent with an old right middle cerebral artery territory infarct

Our patient, the first child of a 37-year-old healthy mother, was delivered at 41 + 3 weeks via a normal vaginal delivery following an uncomplicated gestational period. She had a normal birth weight (3540 g) and exhibited a full Apgar score. Upon discharge on the second post-delivery day, no abnormalities were noted. There was no maternal or family history of autoimmune disease, coagulation disorders, or cerebrovascular events. At 3.5 months of age, the parents observed reduced spontaneous movement in the left limbs. The patient was referred to a physiotherapist and a chiropractor for an initial evaluation, which confirmed reduced spontaneous movement in the left limbs. At 4.5 months of age physical examination revealed normative size for her age, but the neurological examination suggested a left-sided hemiparesis. A cerebral MRI performed at 5 months of age revealed encephalomalacia in the right frontal, temporal, and parietal lobes, consistent with an old right middle cerebral artery infarct (Fig. 1).

Thrombophilia screening performed at the age of 7.5 months showed normal blood count, prothrombin time, activated partial thromboplastin time, antithrombin III, protein C, protein S, and a normal genotype for factor V Leiden and factor II/prothrombin allele, as well as negative results for LA. However, high levels of IgG type aCL and aβ2GPI antibodies were detected (Table 3). The patient’s mother, who is entirely healthy with no prior history of fetal loss or thromboembolic events, tested negative for aPL antibodies 4 weeks after the initial positive aPL test in the patient (Table 3).

**Table 3:** The results of the aPL and LA testing of the patient and mother

	Maternal	Infant						Upper cut-off (99% percentile)
Age (months)		7.5	11	18	32	39	84	
Months after diagnosis of APS	1	0	3	10	24	31	76	
Lupus anticoagulant	Negative	Negative	Negative	-	-	-	Negative	0
Cardiolipin IgG (GPL-U/mL)	1	62	42	9	2	4	2	< 21
Cardiolipin IgM (MPL-U/mL)	18	1	3	2	1	1	3	< 41
Beta-2-glycoprotein IgG (AU/mL)	1	119	46	22	3	2	1	< 11
Beta-2-glycoprotein IgM (AU/mL)	4	< 3	< 3	< 3	< 3	< 3	< 3	< 1 3

Follow-up testing of the patient 3 months later showed persistence of IgG aβ2GPI antibodies and IgG aCL antibodies though at reduced titers confirming infantile APS. The patient exhibited no other systemic manifestations of APS, except for neurological involvement. Both aCL and aβ2GPI antibodies were normalized 2 years after the initial assessment, also confirmed by consecutive negative tests for aPL almost 3 years apart (Table 3).

The patient received antiplatelet therapy with acetylsalicylic acid (ASA) at a dosage of 3 mg/kg, which was maintained until the normalization of high aPL levels, within 2.5 years.

### Patient outcome

Throughout the 5.5-year follow-up period, the patient remained negative for aPL and LA and did not experience any recurrence of thrombotic events after discontinuation of ASA therapy. At the age of six, 8 months before the last normal aPL control, she however experienced focal seizures and was put on antiseizure medication. The follow-up MRI, conducted 6.5 years after the initial scan, revealed stable, extensive polycystic encephalomalacia in the right hemisphere, with no evidence of new infarcts. Additionally, two small hemosiderin deposits were noted at the frontal margin of the right frontal cyst.

## Results

Including the present study, a total of 20 cases of infantile thrombosis associated with de novo synthesis of antiphospholipid antibodies (aPL) were identified across ten articles published as case reports or case series between 2010 and 2023 [5, 7, 8, 9–14] (Table 2).

### Thrombotic manifestations of cases with de novo infantile APS

Among the 20 cases, 14 patients experienced ischemic stroke. One patient presented with small bowel infarction, renal vein thrombosis, and stroke associated with catastrophic APS. Another presented with a right atrial thrombus, while one developed sinus venous thrombosis. Additionally, one patient had deep vein thrombosis (DVT) in the left lower extremity, and another experienced multiple thrombosis in the central nervous system (CNS), affecting cerebral veins, sinuses, and arteries, including the left internal carotid. One further patient exhibited multiple thrombotic events in the context of catastrophic APS (Table 2).

### Prenatal, maternal, and thrombophilic risk factors as “second-hit” mechanisms in infantile APS

Out of the 20 cases analyzed, only two cases (#7 and #11) and the present study (#20) reported no additional prenatal, maternal, or thrombophilic risk factors for thrombosis beyond APS. However, both cases (#7 and #11) were part of a case series with incomplete data. Specifically, case #7 lacked information on thrombophilia risk factors in the infant, while case #11 did not provide details on maternal risk factors (Table 2).

Among the 20 cases, prenatal risk factors for infantile thrombosis included infection or sepsis (7/20), the presence of a central venous catheter (2/20), dehydration (1/20), asphyxia (3/20), prematurity (5/20), respiratory distress syndrome (4/20), fetal inflammatory response syndrome (1/20), residual valvulopathies (1/20), chylothorax (1/20), hypoxic-ischemic encephalopathy 1/20), acute kidney injury (1/20), and macrosomia (1/20). 

Maternal risk factors comprised diabetes or gestational diabetes (3/20), recurrent pregnancy losses or intrauterine fetal death (2/20), thrombus in the umbilical artery or vein (2/20), maternal infection (1/20), twin pregnancy (1/20), placental abruption (1/20), and protein S deficiency (1/20).

Thrombophilic risk factors identified in the infants included heterozygosity for the Factor V (FV) Leiden mutation (3/20), heterozygosity for the prothrombin (20210G/A) mutation (2/20), homozygosity for the methylenetetrahydrofolate reductase (MTHFR) mutation (either 677 T/T or 1298C/C) (3/20), homozygosity or compound homozygosity for plasminogen activator inhibitor-1 (PAI-1) mutations (either 844A/A and 675 4G/4G or 675G/A) (3/20), and elevated lipoprotein (a) levels (1/20) (Table 2).

### Anticoagulation treatment of patients with de novo infantile APS

Data were missing for eight patients. Among the remaining cases, four patients were treated with ASA, one patient received both low-molecular-weight heparin (LMWH) and ASA, one patient was initially given unfractionated heparin, which was later switched to LMWH, three patients were treated with LMWH, and three patients did not receive any anticoagulation therapy (Table 2).

## Discussion

In this paper, we present a case of infantile APS suffered from a neonatal thrombotic stroke, demonstrating persistent positivity for aPL antibodies, particularly high levels of aCL and aβ2GPI antibodies, leading to a diagnosis of primary APS with the absence of other APS manifestations. The mother showed no signs of aPL antibodies, suggesting primary de novo infantile aPL synthesis in the infant. Most notably the patient did not have additional risk factors beyond aPL to trigger de novo primary APS and exhibited a gradual disappearance of aPL over time. We also conducted a review of the existing literature on additional risk factors, termed the “second-hit” mechanism, in reported cases of de novo infantile APS. We identified 20 cases across 10 articles (Table 2). Among these, our case (#20) is the only one without additional prenatal, maternal, or thrombophilic risk factors, based on the 18 cases with complete data reporting all the aforementioned risk factors.

Despite the low incidence of clinical APS manifestations in infants, the brain is the most affected organ by thrombotic events, with the cerebral artery being the most affected site within the brain [6], as also highlighted in our current review. Of the 20 cases included in our review, 10 involved cerebral artery stroke, accounting for the majority of the 16 ischemic stroke cases. A meta-analysis identified the presence of aPL antibodies as a significant risk factor for arterial ischemic strokes in children, including neonates [17]. While there is strong evidence suggesting that aPLs can be pathogenic rather than merely a serological marker, it remains unclear whether aPLs alone are capable of triggering thrombosis.

Autoimmune diseases are uncommon in newborns due to immature immune systems, typically maturing by age 3 [18]. While most cases arise from transplacental transmission of maternal diseases, some pregnancies involve the transmission of aPL without resulting in clinical manifestations of APS. This is supported by a 5-year prospective study of 134 children born to mothers with APS found no instances of thrombosis despite the persistent presence of LA, aCL, or aβ2GPI antibodies) [19]. This further indicates that factors beyond aPL may be required to induce thrombotic events in neonates, supporting the notion of a “second-hit” model.

Given the rarity of infantile APS, only a limited number of cases have been reported, with the majority originating from the transplacental transmission of maternal antibodies. In the present paper, we expanded the previous review conducted by Gordon et al. [7], which included 11 cases of de novo neonatal APS. Our literature review identified 20 cases of de novo infantile APS, based on the criteria outlined in the Materials and Methods. Notably, in 18 of these cases, where complete data were provided by the authors, additional thrombotic risk or trigger factors were identified, with the exception of our case, where no supplementary risk factors were present.

According to a review by Gordon et al. [7] in de novo neonatal APS, the diagnosis was delayed compared to maternally transmitted APS, with an average diagnosis mean time of 4.7 months (range, 5 days to 13 months) after birth versus 2.2 months (range, immediately after birth–14 months). This delay is attributed to low clinician awareness or due to the absence of maternal or family history in de novo cases, whereas in maternally transmitted APS, nearly 40% of mothers had a diagnosis of APS before delivery, and others had a history of multiple miscarriages. In our case, the initial diagnosis of APS was made at 7.5 months after the birth, in a similar range to previously published de novo cases.

Contrary to the “second-hit” model, our case did not exhibit additional risk/trigger factors, typically associated with the onset of thrombotic events in neonatal APS, as recognized in the literature. These diverse risk factors encompass maternal, infant-specific conditions, and hereditary thrombophilia as presented in Table 1 [4, 5, 7–14]. Especially, hereditary thrombophilia, such as low levels of antithrombin, protein C, and protein S, as well as mutations in factors V and II (prothrombin) genes, are associated with thrombotic events in newborns, reflecting an imbalance between coagulants and anticoagulant activity. This is primarily attributed to the characteristics of the neonatal hemostasis system, which is marked by a prothrombotic state [8].

Remarkably, our case stands out as a rare instance of de novo APS without the presence of any other trigger factors, which is unprecedented in the literature. The question arises: what is the mechanism behind this? Berkun et al. [5] speculated that environmental factors, including vaccinations and drugs, might potentially trigger APS. They also proposed that the stress of delivery might induce the de novo aPL development in neonates.

Much of the information regarding the treatment of infantile APS is lacking in the reported cases in the literature [4–7] as also confirmed in our review (Table 2). The optimal therapy for infantile APS remains debated, with management primarily relying on observational studies and clinical expertise, leading to varied treatment approaches [20]. In our case, considering the patient’s age of 8 months and the absence of additional trigger risk factors, we chose antiplatelet therapy with a low dose of ASA (3 mg/kg/day) treatment which was continued until the normalization of aPL levels. Notably, Berkun et al. reported that high aPL levels normalized in 10 out of 12 neonatal APS cases with stroke, similar to our case, within 2.5 years [5]. Pines M et al. [14] described two de novo infantile APS cases—one with an atrial thrombus and another with multiple CNS thromboses—where LMWH was continued until aβ2GPI antibodies normalized at 37 and 59 weeks. During follow-up, no further thrombotic events were reported after 3 and 7 years following the cessation of anticoagulation. They suggested sequential aPL testing to guide treatment. These findings suggest that some patients meeting APS criteria may experience transient disease without recurrence, potentially eliminating the need for long-term anticoagulant therapy unless other risk factors are present. It is tempting to define these patients as having “pseudo infantile APS”. However, further longitudinal and larger cohort studies are necessary to gain a deeper understanding of this phenomenon.

A key strength of this study is its novelty, as it represents the first case report and review to specifically focus on infantile de novo APS and the identification of additional triggering risk factors, as a “second-hit mechanism” observed in affected patients. One limitation of our report is the timing of aPL testing in the mother, which was conducted one month after the patient’s APS diagnosis. This issue was also observed in most of the cases presented in Table 2, where aPL testing in the mother and infant was not performed simultaneously. Although transient aPL positivity during pregnancy cannot be completely excluded, it is considered unlikely in our case. The mother is healthy, with no history of fetal loss or thromboembolic events, and she experienced a normal vaginal delivery following an uncomplicated pregnancy. Another limitation of our study is the potential for incomplete patient data in our review, as outlined in Table 2, particularly when patients were reported within case series rather than as individual case reports.

In conclusion, this case presents a unique instance of de novo infantile APS characterized by the absence of additional trigger risk factors as a “second hit” among the 18 cases of de novo neonatal APS with complete data according to our review of cases reported in the literature. Moreover, the patient’s condition exhibited an atypical course, marked by the absence of other APS manifestations and a gradual disappearance of aPL. This unique case calls for further investigation into the mechanisms underlying infantile APS, particularly the potential role of environmental factors in initiating de novo antibody synthesis. Additionally, it raises questions about the necessity of long-term anticoagulation therapy in cases where no triggering risk factors are present.

## Data Availability

No datasets were generated or analysed during the current study.
